# Asthma diagnosis and treatment – 1019. Churg Strauss Syndrome – missed diagnosis and consequences

**DOI:** 10.1186/1939-4551-6-S1-P19

**Published:** 2013-04-23

**Authors:** Mahomed Cassim Kamdar

**Affiliations:** 1 Medicine and Pulmonology, Nelson Mandela School of Medicine University of KwaZulu Natal/ RK Khan Hospital, South Africa

## Background

To have a high index of suspicion for diagnosing Churg Strauss Syndrome in patients presenting with asthma symptoms, pulmonary infiltrates and various systemic manifestations of the disease.

## Methods

Case notes with investigations was retrieved from various specialists attending to the index patient with undiagnosed pulmonary symptoms and pulmonary infiltrates withsystemic disease,especially chronic abdominal symptoms over a two year period. During the patient's last admission she again presented with intractable abdominal pain, pyrexia, systemic hypertension (BP 170/140), loss of weight, emaciated (24 Kg), purpuric eczematous rash on the upper limb,unable to walk with marked weakness (polyneuropathy) and was being prepared for laparoscopic cholecystectomy by the surgeons for suspected cholecystitis.

## Results

Review of data and clinical features revealed a classical case of Churg Strauss Syndrome, highlighting the phasic nature of the disease.Beginning with the prodromal allergic phase progressing to the eosinophilic phase, vasculitic phase and post-vasculitic phase.

## Conclusions

A classic case of undiagnosed Churg Strauss Syndrome over a 2-year period highlighting the sequential x-ray chest, CT, histological features (endoscopy and abdominal laparoscopy) and the systemic manifestation of the disease with a brief review of the topic. Suitable for 20 or 30 minutes oral presentation.

